# Prospective descriptive pilot feasibility case series of low-pressure cataract surgery with the Zeiss Quatera 700 in glaucoma patients

**DOI:** 10.3389/fopht.2026.1824059

**Published:** 2026-09-24

**Authors:** Koen Willekens

**Affiliations:** Visionair oogzorg, Geel, Antwerp, Belgium

**Keywords:** cataract surgery, fluidics, glaucoma, low pressure cataract surgery, ocular perfusion

## Abstract

**Purpose:**

The aim of this study was to evaluate the feasibility of low-pressure cataract surgery using the Zeiss Quatera 700 system in patients with glaucoma.

**Patients and methods:**

This is a prospective, investigator-initiated case series of 10 eyes of 10 patients with glaucoma undergoing cataract surgery with the Quatera 700. The minimal intraocular pressure (IOP) settings (30 mmHg) were used for every step of the surgery. The primary outcome was defined as the occurrence of any surgical complication. All other outcome variables were prospectively classified as secondary and exploratory endpoints. Secondary outcomes included intraoperative parameters like phaco energy usage, postoperative IOP spikes (>30 mmHg), and visual function [best-corrected visual acuity (BCVA) and visual field mean deviation (MD)].

**Results:**

All surgeries were completed without intraoperative complications (0.0% event rate; exact 95% Clopper–Pearson CI: 0.0% to 30.8%). Mean effective phaco time was 3.24 s (± 2.01 s). Postoperative IOP spikes (>30 mmHg) occurred in two patients (20.0% event rate; exact 95% Clopper–Pearson CI: 2.5% to 55.6%) and were managed with additional acetazolamide. BCVA improved in 9 of 10 eyes (exact 95% CI: 55.5%, 99.8%). The median paired logMAR change from preoperative baseline to the 1-month postoperative follow-up was −0.10 logMAR (range: −1.30 to +0.10 logMAR, *p* = 0.0137), and visual field MD remained stable at 1 month postoperatively.

**Conclusion:**

This pilot study demonstrates that low-pressure cataract surgery at a target setting of 30 mmHg using the Zeiss Quatera 700 is clinically feasible in patients with glaucoma. Given the small sample size and short follow-up of this pilot series, larger randomized comparative trials are required to confirm these preliminary safety profiles and determine long-term clinical benefits in a broader glaucoma population.

## Introduction

Cataract surgery in patients with glaucoma presents unique challenges. Intraoperative elevation of intraocular pressure (IOP), often reaching 60–90 mmHg (81–122 cmH2O) with conventional phacoemulsification systems, can transiently reduce ocular perfusion, posing a risk to the already compromised optic nerve in patients with glaucoma (1, 2). Preserving ocular perfusion during surgery is critical for preventing further glaucomatous damage.

Recent studies have investigated the impact of low IOP during cataract surgery and have shown promising results for safety and endothelial outcomes, especially in vulnerable populations (3). Beres et al. (2022) reported that low-pressure microincision cataract surgery resulted in fewer surge events and more stable anterior chamber dynamics compared to standard infusion pressures (4).

The Zeiss Quatera 700 (Carl Zeiss Meditec, Jena, Germany) cataract surgery system introduces a novel fluidic platform, the QUATTRO Pump. Unlike peristaltic or Venturi systems, the QUATTRO Pump is a synchronized dual-pump system capable of real-time infusion and aspiration balance. This results in increased anterior chamber stability, even at lower target IOPs of 30 mmHg (5). Because of the QUATTRO Pump system, surge duration has been reduced significantly compared to conventional pumps. Leakage compensation and reduced passive vacuum due to the use of large bore tubing result in a stable effective IOP (± 2 mmHg around target IOP). Conventional phaco systems require higher infusion pressures to compensate for surge and aspiration variability. A stable anterior chamber with a near-physiological IOP could also improve comfort for patients undergoing surgery under topical anesthesia. Liu et al. (2023) found significantly lower pain levels among patients undergoing surgery with active fluidics with a target IOP of 30 mmHg compared to those undergoing surgery with the gravity fluid system with a target IOP at 80 cmH_2_O (=59 mmHg) (6).

This study evaluates the feasibility of using low IOP settings during cataract surgery with the Zeiss Quatera 700 in patients with glaucoma.

## Methods

This prospective, observational, single-center, pilot feasibility case series was conducted between October 2024 and June 2025. Ten eyes of 10 patients with glaucoma scheduled for cataract surgery were included. This cohort size was a pragmatic sample for this initial technical feasibility series. This study is not powered to estimate uncommon complications or establish comparative safety. In standard phacoemulsification, the baseline average rate of severe intraoperative complications—such as posterior capsule rupture (PCR)—is low, typically ranging between 0.6% and 2.1% in modern large-scale ophthalmic databases (7, 8) Applied to this small sample size, the statistical “Rule of Three” provides an approximate upper bound of 30%, whereas the exact Clopper–Pearson 95% upper confidence limit dictates that if zero serious adverse events (SAEs) occur across these 10 eyes, we can state with 95% confidence that the true risk of an SAE under low-pressure parameters is less than 30.8% (9).

All surgeries were performed by a single surgeon using the Zeiss Quatera 700 system. Eyes from patients with endothelial dystrophy and active uveitis, known corticosteroid responders, or functionally monophthalmic patients were excluded. The study was approved by the ethical review committee at the University of Antwerp (UA) and was conducted in accordance with Good Clinical Practice within the tenets of the Declaration of Helsinki (registered in clinicaltrials.gov as NCT06712758). All patients signed informed consent and received cataract treatment as standard of care. Preoperative measurements included best-corrected visual acuity (BCVA), IOP, visual field mean deviation (VF MD) (Humphrey Field Analyzer 840, Carl Zeiss Meditec, Jena, Germany), average retinal nerve fiber layer (RNFL) thickness and vertical cup–disc ratio (VCDR) (Cirrus 500 OCT, Carl Zeiss Meditec, Jena, Germany), endothelial cell count (Perseus, CSO, Italy), axial length, anterior chamber depth (ACD), and central corneal thickness (CCT) (IOLMaster 700, Carl Zeiss Meditec, Jena, Germany), along with demographic information (age, gender, medical history, type of glaucoma, and current medication).

### Surgical technique

All surgeries were performed using the Zeiss Quatera 700 system. The settings were standardized as depicted in **Table 1**.

**Table 1:** Surgical parameters.

	IOP (mmHg)	Vacuum (mmHg)	Dynamic factor	Longitudinal US power	
Quadrant removal soft	30	40–650 (linear)	Fastest	Linear 0–40, pulse	
Quadrant removal hard	30	40–650 (linear)	Fastest	Linear 0–40, APM	
I/A cortex removal	30	0–650 (linear)	Fast	NA	
I/A visco removal	30	0–650 (linear)	Fast	NA	

APM, advance power modulation and US, ultrasound.

After applying topical anesthesia (oxybuprocaine hydrochloride 0.4%, Bausch and Lomb, UK Ltd., Kingston-upon-Thames, UK) and positioning the patient on the surgical table, a time-out procedure was performed. The eye was disinfected with Iso-Betadine 5% (Mylan, Canonsburg, PA, USA) and a sterile cover was placed. An eyelid speculum was inserted and a second application of iso-betadine was carried out before rinsing with a balanced salt solution and starting the surgery. First, the main incision was made with a 2.2-mm blade (Surgicon AG, St Gallen, Switzerland) either superior or temporal depending on the steep axis of the cornea. Mydrane was injected into the anterior chamber to dilate the pupil, followed by stabilization of the anterior chamber through the injection of visco-elastic (Z-hyalin, Carl Zeiss Meditec, Jena, Germany) with digital monitoring of the IOP to maintain IOP within the physiologic range. Afterward, two side ports of 1.2 mm (Surgicon AG, St Gallen, Switzerland) were created 90° from the main port, and an incision was made in the anterior lens capsule. A rhexis of 5-mm diameter was made with a forceps (Blink Medical Ltd, Shirley, United Kingdom) before performing a hydrodissection. The anterior chamber was again filled with methylcellulose (Z-celcoat, Carl Zeiss Meditec, Jena, Germany) to allow pre-chopping (Janach srl, Como, Italy) of the lens nucleus. Afterward, the phaco handpiece with continuous infusion activated was placed in the anterior chamber and the lens pieces were aspirated using a dual linear foot control setting. A 21-G straight flared phaco tip was used. The cortex remnants were aspirated using disposable 21-G irrigation and aspiration handpieces (Carl Zeiss Meditec, Jena, Germany), and the capsular bag was polished when necessary. The bag and anterior chamber were filled with visco-elastic, and the appropriate hydrophobic foldable CT Lucia IOL (Carl Zeiss Meditec, Jena, Germany) was placed in the bag. The visco-elastic was removed with I/A handpieces, and the corneal side ports were irrigated. To conclude the surgery, 0.05 cc of cefuroxime (Aprokam, Thea Pharma, Clermont-Ferrand, France) was injected into the anterior chamber.

Postoperative follow-up consisted of visits on day 1, week 1, and week 4 after surgery. Visual field tests and Optical coherence tomography (OCT) imaging were repeated on week 4.

### Outcome measures

The primary outcome of this investigation was defined as a binary safety event (the occurrence of any intraoperative surgical complication), evaluated using a two-stage validation protocol. The first stage involved recording of required changes in the aspiration or infusion settings differing from those mentioned in **Table 1**, direct intraoperative clinical logging by the operating surgeon, including the noted utilization of specific procedural adjuncts [capsular tension rings, vitrectomies, carbachol intraocular solution (Miostat), Malyugin rings, or iris hooks], rescue maneuvers, and possible malfunctioning or error messages during priming or surgery of the Quatera’s software.

The second stage consisted of a retrospective postoperative video analysis by the surgeon of the entire procedure to record total duration and identify transient structural deviations, specifically excluding or identifying moments involving a shallow anterior chamber, iris prolapse, or capsular protrusion in front of the iris plane, in addition to all criteria mentioned in the first stage of the validation protocol. All continuous clinical parameters and intermediate post-surgical evaluation timelines were prospectively classified as secondary and exploratory endpoints. These secondary outcomes consist of BCVA, IOP, MD, and RNFL thickness change at month 1 together with effective phaco time (EPT) and total duration of the surgery. The primary endpoint was defined prior to the analysis of the study results. During the revision process, the definition of an IOP spike as an IOP >30 mmHg at any given time point was added, in order to provide a clear and consistent definition for the study results analysis.

### Statistical analysis

The primary outcome of this investigation was defined as a binary safety event, evaluated using a two-stage validation protocol that paired direct intraoperative clinical logging with retrospective postoperative video analysis. Binary primary and secondary parameters were expressed as frequencies and percentages, with corresponding exact 95% confidence intervals (CIs) calculated via the Clopper–Pearson exact binomial distribution method to accommodate small sample sizes.

All continuous parameters and intermediate post-surgical evaluation timelines were prospectively classified as secondary and exploratory. Continuous variables were summarized as mean ± standard deviation (SD) for normally distributed data, or as median with minimum and maximum values for data deviating from a normal distribution. Categorical variables were expressed as frequencies and percentages. The assumption of normality for all continuous paired data differences was evaluated visually via quantile–quantile (Q–Q) plots, and evidence of departure from normality was identified using the Shapiro–Wilk test. For the Wilcoxon signed-rank analysis of BCVA, zero differences and ties were handled using Pratt’s method to preserve the structural information of tied ranks, and the resulting *p*-value was calculated as an asymptotic approximation using the scipy.stats.wilcoxon function in Python.

Changes from baseline to the final postoperative follow-up for normally distributed continuous metrics were analyzed using the two-tailed paired *t*-test. For continuous parameters that significantly violated normality assumptions, the non-parametric Wilcoxon signed-rank test was applied.

No multiplicity adjustment was applied because all secondary analyses were exploratory. The reported *p*-values are thus nominal and hypothesis-generating. Statistical significance was set at *p* < 0.05. Statistical analyses were performed using the Python programming language (Version 3.10.12). The core computational environment was managed within a standardized Unix-based runtime container to ensure cross-platform consistency. Data structures and basic descriptive statistics were handled via Pandas (Version 2.1.4) and NumPy (Version 1.26.2). Hypothesis testing and normality validations were executed using SciPy (Version 1.11.4). To guarantee algorithmic replication, all stochastic and ranking metrics were calculated without arbitrary randomization state overrides.

The core source code, including the primary statistical models and Q–Q plots, is available as **Supplementary Material**.

## Results

The baseline characteristics of the study cohort are compiled and displayed according to their distribution type in **Table 2**. Ten eyes of 10 patients with glaucoma were included. Nine patients were taking IOP-lowering drops with a median of 2.0 (range, 1.0 to 3.0) medications per day. The average baseline ACD was 3.06 ± 0.42 mm. There were two eyes (20.0%) with a history of primary angle closure glaucoma (PACG) and eight eyes (80.0%) presenting with primary open angle glaucoma (POAG).

**Table 2:** Demographic and baseline parameters (*N* = 10).

Parameter	Outcome
Gender (male/female), *n* (%)	5 (50.0%)/5 (50.0%)
Eye (right/left), *n* (%)	6 (60.0%)/4 (40.0%)
Age (years), mean ± SD	71.50 ± 8.50
Visual field MD (dB), mean ± SD	−4.79 ± 5.46
Axial length (mm), mean ± SD	23.83 ± 1.12
ACD (mm), mean ± SD	3.06 ± 0.42
CCT (μm), mean ± SD	555.80 ± 44.77
ECD (cells/mm²), mean ± SD	2,724.89 ± 316.79
Preoperative BCVA (LogMar), median (min–max)	0.13 (0.00–1.30)
Number of topical IOP lowering medication, median (min–max)	2 (0–3)
VCDR (%), mean ± SD	69.2% ± 11.5%
IOL power (diopter), mean ± SD	21.65 ± 3.25

MD, mean deviation; ACS, anterior chamber depth; CCT, central corneal thickness; ECD, endothelial cell density; BCVA, best-corrected visual acuity; VCDR, vertical cup–disc ratio; and IOL, intraocular lens.

Per the primary protocol, the intraoperative complication rate was 0.0% (0 out of 10 eyes), with an exact 95% CI of 0.0% to 30.8%. Retrospective video reviews confirmed that no eyes required capsular tension rings, vitrectomies, Miostat, Malyugin rings, or iris hooks. The mean duration of surgery was 443 ± 98 s, with no recorded instances of shallow anterior chamber, iris prolapse, or capsular protrusion in front of the iris plane. Postoperative safety profiles, tracking acute IOP spikes (*post-hoc* definition applied to prospectively collected IOP measurements; IOP > 30 mmHg), revealed an incidence rate of 20.0% (2 out of 10 eyes; exact 95% CI: 2.5% to 55.6%). These events occurred in Patient Q3 (delayed spike of 33.4 mmHg at week 1) and Patient Q7 (acute spike of 35.0 mmHg on day 1). Both cases resolved within 48 h following short-term systemic acetazolamide administration.

The continuous outcomes matrix tracking baseline to 1-month changes is reported in **Table 3**. Visual inspection of the Q–Q plots and the Shapiro–Wilk test did not identify strong evidence of departure from normality, except for the paired differences for BCVA.

**Table 3:** Preoperative to 1-month postoperative clinical outcomes.

Clinical metric	Baseline summary	Postoperative summary	Paired change	95% CI or IQR of change	Statistical test	Nominal *p*-value
IOP (mmHg), mean ± SD	18.65 ± 6.79	14.79 ± 2.46	−3.86 ± 6.43	[−8.46, +0.74]	Paired *t*-test	0.0901
BCVA (logMAR), median (IQR) [range]	0.12 (0.12) [0.00, 1.30]	0.00 (0.05) [0.00, 0.15]	−0.10 (0.16) [−1.30, +0.10]	IQR: 0.16 Range: [−1.30, +0.10]	Wilcoxon signed-rank	0.0137
VF MD (dB), mean ± SD*	−4.74 ± 5.78	−4.16 ± 4.39	+0.58 ± 3.14	[−1.83, +3.00]	Paired *t*-test	0.5926
Average RNFL thickness (μm), mean ± SD	72.57 ± 11.55	76.59 ± 11.59	+4.02 ± 5.04	[+0.42, +7.63]	Paired *t*-test	0.0324
Spherical equivalent (SE, D), mean ± SD	−1.07 ± 3.55	−0.25 ± 0.85	+0.82 ± 3.22	[−1.48, +3.13]	Paired *t*-test	0.4392

BCVA, best-corrected visual acuity; IOP, intraocular pressure; D, diopter (D); MD, mean deviation; RNFL, and retinal nerve fiber layer (RNFL).

*1 missing value of VF MD at month 1.

At 1-month postoperatively, mean IOP decreased from 18.65 ± 6.79 mmHg to 14.79 ± 2.46 mmHg, representing a mean paired reduction of −3.86 mmHg (95% CI: −8.46 to +0.74 mmHg; *p* = 0.0901). **Figure 1** depicts all IOP measurements. BCVA improved in 90.0% of eyes (9 of 10 eyes; exact 95% CI: 55.5%, 99.8%). The median paired logMAR change from preoperative baseline to the 1-month postoperative follow-up was −0.10 logMAR (range: −1.30 to +0.10 logMAR, *p* = 0.0137). Structural monitoring revealed an increase in average measured RNFL thickness (+4.02 μm; 95% CI: +0.42 to +7.63 μm; *p* = 0.0324), while functional VF MD values remained stable with a mean paired shift of +0.58 dB (95% CI: −1.83 to +3.00 dB; *p* = 0.5926). Evaluation of scan diagnostics revealed a highly significant improvement in paired OCT signal strength, rising from a baseline mean of 6.50 ± 1.43 to a postoperative mean of 8.80 ± 0.92 (mean change: +2.30; 95% CI: +1.35 to +3.25; *p* < 0.001).

**Figure 1:**
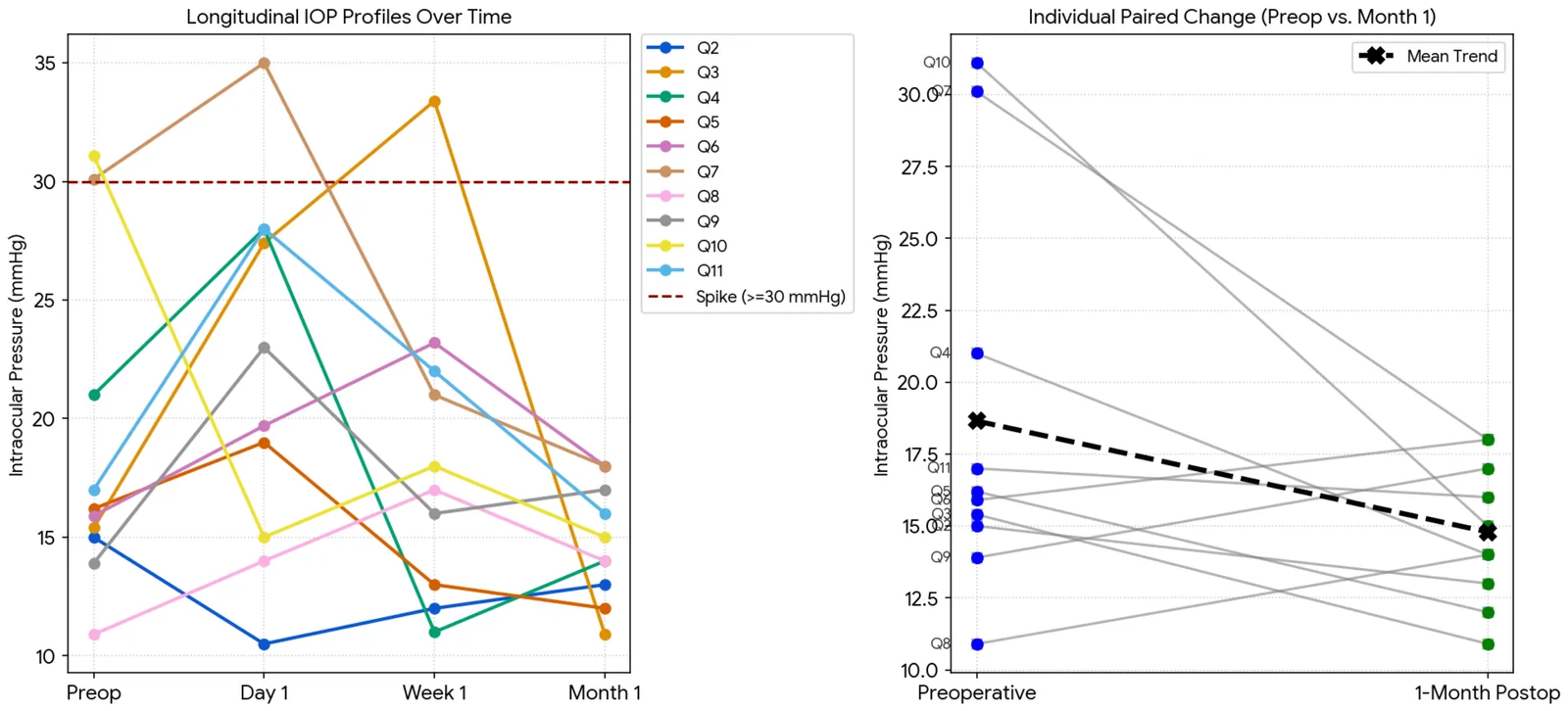
Longitudinal intraocular pressure (IOP) measurements.

## Discussion

### Safety and feasibility of low-pressure cataract surgery (IOP 30 mmHg)

All 10 procedures were completed without any observed intraoperative complication. The small sample provides limited precision for estimating actual complication rates and is underpowered for a safety claim, but this study shows that performing a low-pressure cataract surgery with Quatera 700 is feasible.

Notably, the anterior chamber remained stable even during critical moments such as phaco tip occlusion and sudden surge. Conventional phaco machines, relying on gravity fluid systems, frequently require higher infusion pressures (60 mmHg or more) to counteract a dangerously low anterior chamber volume during surge, especially when working with high vacuum aspiration. Zanini et al. (2003) stated that in order to be able to work with higher vacuum levels, the bottle height should be increased (10). To decrease the unwanted passive vacuum and the extent of surge, the inner diameter of the aspiration line is decreased by some manufacturers in order to increase resistance and decrease the aspiration flow rate. The necessity of supraphysiological IOPs during conventional phaco surgery exposes the optic nerve and trabecular meshwork to transient ischemic episodes, particularly detrimental in patients with glaucoma and compromised ocular perfusion pressure (2). Ramsden et al. (2023) found that a complete wipe-out is rare but occurs predominantly in advanced glaucomatous eyes (11). The core advantage of the QUATTRO Pump lies in its ability to directly synchronize infusion and aspiration through a dual-cylinder mechanical pump, compensating in real time for leakage. Unlike traditional peristaltic, Venturi, or even active fluidics bag systems (e.g., Centurion, Stellaris Elite), which attempt to compensate for outflow via passive or semi-active means, the Quatera continuously calculates and adjusts fluid flow based on incision leakage, predicted occlusion breaks, and sudden changes in aspiration demand. Crucially, clinicians must clearly distinguish the target machine setting from the true, measured IOP. Target IOP is the digital fluidic parameter programmed into the phacoemulsification console, whereas measured (effective) IOP is the true, dynamic hydrostatic pressure inside the patient’s anterior chamber during active surgery. In standard platforms, high fluidic demand or an occlusion break causes a large discrepancy between these two metrics, frequently creating unmeasured hypotony or post-occlusion surges that collapse the anterior chamber. Bench-testing data demonstrate that the Zeiss Quatera 700 minimizes this fidelity gap via its proprietary, sensor-driven QUATTRO Pump, which directly controls fluid volume to maintain the effective IOP at its programmed target up to an aspiration flow rate of 90 cc/min. Furthermore, at a highly restricted low-pressure target setting of 30 mmHg, the platform successfully mitigates fluidic fluctuations by bounding post-occlusion surge durations to a brief 282 to 408 ms, ensuring that the target setting and the patient’s effective intraoperative pressure remain highly congruent—retaining variances strictly within 2.2 mmHg of the target IOP (5).

Beres et al. (2022) showed that a low-infusion approach during microincision cataract surgery improves intraoperative chamber stability (4). Furthermore, Rauen and colleagues (2024) demonstrated that maintaining IOPs at or below 30 mmHg with active fluidics significantly reduced corneal trauma, inflammation, and endothelial cell loss compared to conventional high-pressure techniques (12).

The concept of lowering intraoperative IOP is increasingly supported by comparative studies showing better chamber stability, fewer complications, and sustained postoperative IOP benefits (13, 14). In particular, patients with pseudoexfoliation syndrome and angle-closure glaucoma have shown favorable pressure control outcomes post phaco-emulsification (15).

Moreover, intraoperative variables such as aspiration pressure, total ultrasound time, and incision architecture have been shown to impact long-term IOP response, underscoring the importance of optimizing phaco parameters when targeting low-pressure surgery (16). Wang et al. (2025) demonstrated that using the Centurion Active Fluidics with active sentry handpiece at an IOP of 20 mmHg provided good chamber stability, absence of complications, and a significant reduction in surges (17). The lowest possible target IOP setting in the current Quatera 700 software is 30 mmHg. Considering the reassuring experiences of others operating at even lower pressures, efficient cataract extraction with a stable anterior chamber at physiologic (± 15 mmHg) IOPs might be possible in the future.

### Efficiency: low phaco energy and short surgical times

Our data revealed an EPT of 3.24 ± 2.01 s. This is not easily comparable because of the small sample size, the lack of a cataract grading system, and the lack of a control group in this study. However, lower IOPs were associated with decreased fluid consumption and reduced stress on corneal endothelial cells, as shown by Rauen et al. (2024). Their study confirms that low-pressure settings can optimize both safety and efficiency, supporting our findings of short surgical duration and excellent visual recovery. Lower ultrasound energy reduces thermal injury risk, corneal endothelial cell loss, and postoperative inflammation (18). Pre-chopping also contributes to the lower overall EPT obviating the need for sculpting.

### Functional outcomes

Our findings demonstrated that most patients experienced excellent visual outcomes, often without the need for additional refractive solutions. All received a white hydrophobic Zeiss CT Lucia IOL. Our findings of improved visual outcomes and modest IOP reduction are consistent with prior reports showing long-term IOP reduction following phacoemulsification, including those using femtosecond-assisted platforms (19, 20). As with other studies involving patients with glaucoma, a transient IOP elevation was noted on the first postoperative day, likely due to retained viscoelastic, inflammation, or trabecular meshwork debris (21). Importantly, this spike was within a manageable range (21.96 ± 7.68 mmHg) and only two patients needed additional systemic acetazolamide for 2 days. The MD did not change significantly, but the average RNFL thickness increase occurred alongside a marked improvement in OCT signal strength and is therefore likely to reflect, at least partly, an improved image acquisition after cataract removal rather than true neural tissue change. This was also reported previously by Mwanza et al. (2011) (22).

### Limitations

This study has several limitations that should be considered when interpreting our findings. First, the sample size was small, consisting of only 10 patients, which restricts the statistical power to detect rarer intraoperative complications or subtle postoperative clinical variations. Second, all surgical interventions were performed by a single surgeon at a single center. While this eliminates inter-surgeon technical variability, it may limit the external validity and generalizability of our surgical techniques to broader ophthalmic practices. Third, our study cohort represents a highly selected population with specific baseline glaucoma profiles, which may not completely reflect the physiological responses of a more heterogeneous surgical population.

Additionally, the study design lacked masking (blinding), which could introduce subtle ascertainment biases during clinical follow-up. Furthermore, the 1-month postoperative follow-up window represents a short timeline that primarily captures acute structural and pressure-related fluctuations. This brief observation period precludes the evaluation of long-term surgical success, delayed complications, or true longitudinal stability of the optic nerve head architecture. Finally, the absence of a parallel control group prevents a direct comparative isolation of phacoemulsification as the sole driver of the observed exploratory secondary changes.

## Conclusion

In conclusion, this pilot study demonstrates that low-pressure cataract surgery at a target setting of 30 mmHg using the Zeiss Quatera 700 is clinically feasible in patients with glaucoma. Given the small sample size and short follow-up of this pilot series, larger randomized comparative trials are required to confirm these preliminary safety profiles and determine long-term clinical benefits in a broader glaucoma population.

## Data Availability

The raw data supporting the conclusions of this article will be made available by the authors, without undue reservation.
