# Supplementary material for: Prospective descriptive pilot feasibility case series of low-pressure cataract surgery with the Zeiss Quatera 700 in glaucoma patients
Source: Front Ophthalmol (Lausanne). 2026 Sep 24;6:1824059. doi: 10.3389/fopht.2026.1824059 (PMC13645598; doi:10.3389/fopht.2026.1824059)

Quatera study statistical analysis Python structure

python

```
# -*- coding: utf-8 -*-
"""
Ophthalmic Surgical Outcomes Analysis Workflow
Archived for Replication, Software Verification, and Transparency
Auditing
"""

import numpy as np
import pandas as pd
import scipy.stats as stats
import statsmodels.stats.proportion as prop
import matplotlib.pyplot as plt

# 1. Dataset Reconstitution from Verified Clinical Matrix

data = {
    'Patient': [f'Q{i}' for i in range(2, 12)],
    'Eye': ['OS', 'OS', 'OD', 'OS', 'OD', 'OD', 'OS', 'OD', 'OD',
'OD'],
    'IOP_pre': [15.0, 15.4, 21.0, 16.2, 15.9, 30.1, 10.9, 13.9, 31.1,
17.0],
    'BCVA_pre': [0.1, 1.3, 0.1, 0.7, 0.15, 0.05, 0.05, 0.15, 0.2, 0.0],
    'VF_MD_pre': [-11.26, -5.18, -16.57, -3.77, -5.03, -5.19, -1.33,
0.25, -0.37, 0.59],
    'RNFL_pre': [57.25, 66.14, 55.4, 82.12, 76.31, 82.72, 71.85, 77.07,
65.51, 91.3],
    'RNFL_post': [69.62, 65.0, 56.7, 88.71, 77.92, 87.49, 73.83, 74.15,
77.22, 95.26],
    'IOP_post_1m': [13.0, 10.9, 14.0, 12.0, 18.0, 18.0, 14.0, 17.0,
15.0, 16.0],
    'BCVA_post_1m': [0.0, 0.0, 0.0, 0.05, 0.0, 0.15, 0.05, 0.05, 0.0,
0.0],
    'VF_MD_post_1m': [-11.25, np.nan, -9.74, -0.55, -7.5, -2.88, -4.75,
-1.05, -0.11, 0.4],
    'SE_pre': [5.75, -1.75, -2.0, -2.5, -1.25, 1.0, 0.0, 1.75, -7.25, -
4.5],
    'SE_post': [0.0, 0.0, 0.0, 0.5, 0.25, -0.75, 0.0, 0.0, -2.5, 0.0]
}

df = pd.DataFrame(data)

# 2. Production Workflow of Visual Q-Q Diagnostic Plots
metrics = {
    'IOP Difference': df['IOP_post_1m'] - df['IOP_pre'],
    'RNFL Difference': df['RNFL_post'] - df['RNFL_pre'],
    'SE Difference': df['SE_post'] - df['SE_pre'],
    'VF MD Difference': (df['VF_MD_post_1m'] -
df['VF_MD_pre']).dropna()
}

fig, axes = plt.subplots(2, 2, figsize=(10, 8))
axes = axes.flatten()

for idx, (label, delta) in enumerate(metrics.items()):
```

```
    stats.probplot(delta, dist="norm", plot=axes[idx])
    axes[idx].set_title(label)
    axes[idx].grid(True, linestyle='--', alpha=0.5)

plt.tight_layout()
plt.savefig('journal_supplementary_qq_plots.png', dpi=300)
print("[SUCCESS] Supplementary Q-Q plots exported as
'journal_supplementary_qq_plots.png'")

# 3. High-Fidelity Replication Statistics Pipeline
print("\n--- Executing Analytic Verification Protocol ---")

# BCVA Wilcoxon Signed-Rank Test with Pratt modification for structural
zeroes/ties
w_stat, w_p = stats.wilcoxon(df['BCVA_pre'], df['BCVA_post_1m'],
zero_method='pratt', correction=True)
print(f"BCVA Change (Wilcoxon Pratt, Asymptotic Normal): Stat =
{w_stat:.1f}, P-value = {w_p:.4f}")

# Parametric Relational Paired t-tests
t_stat_iop, p_iop = stats.ttest_rel(df['IOP_post_1m'], df['IOP_pre'])
print(f"IOP Change (Paired t-test): t = {t_stat_iop:.3f}, P-value =
{p_iop:.4f}")

t_stat_rnfl, p_rnfl = stats.ttest_rel(df['RNFL_post'], df['RNFL_pre'])
print(f"RNFL Change (Paired t-test): t = {t_stat_rnfl:.3f}, P-value =
{p_rnfl:.4f}")
```
Use code with caution.

Comprehensive Data Dictionary and Metadata Structure

To ensure reviewer audits match your institutional logs perfectly, your data repository utilizes the following standardized layout parameters:

Column Header ID	Primary Metric Category	Open-Science Data Type	Computational Scale Constraints	Missing Values Handling
**Patient**	Subject ID Key	String / Nominal	`Q2` to `Q11` (N=10)	None
**Eye**	Surgical Eye Designation	Categorical / Factor	`OD` (Right), `OS` (Left)	None
**IOP_pre**	Baseline Pressure	Float / Continuous	Range: \([10.9, 31.1]\) mmHg	None
**IOP_post_1m**	Postoperative Pressure	Float / Continuous	Range: \([10.9, 18.0]\) mmHg	None
**BCVA_pre**	Baseline Visual Acuity	Float / Continuous	Range: \([0.00, 1.30]\) decimal	None
**BCVA_post_1m**	Postoperative Visual Acuity	Float / Continuous	Range: \([0.00, 0.15]\) decimal	None
**VF_MD_pre**	Baseline Mean Deviation	Float / Continuous	Range: \([-16.57, 0.59]\) dB	None

**VF_MD_post_1m**	Postoperative Mean Deviation	Float / Continuous	Range: \([-11.25, 0.40]\) dB	Q3 dropped due to missing charts
**RNFL_pre**	Pre-op Nerve Thickness	Float / Continuous	Range: \([55.40, 91.30]\) \(\mu \)m	None
**RNFL_post**	Post-op Nerve Thickness	Float / Continuous	Range: \([65.00, 95.26]\) \(\mu \)m	None

Q-Q plots:

1. Intraocular Pressure (IOP) Differences

Shapiro-Wilk Test Validation: W = 0.9181, P = 0.3414 (Normally Distributed)

- **Point 1:** (-1.4988, -16.1000)
- **Point 2:** (-0.9849, -12.1000)
- **Point 3:** (-0.6470, -7.0000)
- **Point 4:** (-0.3711, -4.5000)
- **Point 5:** (-0.1212, -4.2000)
- **Point 6:** (0.1212, -2.0000)
- **Point 7:** (0.3711, -1.0000)
- **Point 8:** (0.6470, 2.1000)
- **Point 9:** (0.9849, 3.1000)
- **Point 10:** (1.4988, 3.1000)

2. Retinal Nerve Fiber Layer (RNFL) Thickness Differences

Shapiro-Wilk Test Validation: W = 0.9342, P = 0.4908 (Normally Distributed)

- **Point 1:** (-1.4988, -2.9200)
- **Point 2:** (-0.9849, -1.1400)
- **Point 3:** (-0.6470, 1.3000)
- **Point 4:** (-0.3711, 1.6100)
- **Point 5:** (-0.1212, 1.9800)
- **Point 6:** (0.1212, 3.9600)
- **Point 7:** (0.3711, 4.7700)
- **Point 8:** (0.6470, 6.5900)
- **Point 9:** (0.9849, 11.7100)
- **Point 10:** (1.4988, 12.3700)

3. Spherical Equivalent (SE) Differences

Shapiro-Wilk Test Validation: W = 0.9357, P = 0.5063 (Normally Distributed)

- **Point 1:** (-1.4988, -5.7500)
- **Point 2:** (-0.9849, -1.7500)
- **Point 3:** (-0.6470, -1.7500)
- **Point 4:** (-0.3711, 0.0000)
- **Point 5:** (-0.1212, 1.5000)
- **Point 6:** (0.1212, 1.7500)
- **Point 7:** (0.3711, 2.0000)
- **Point 8:** (0.6470, 3.0000)
- **Point 9:** (0.9849, 4.5000)
- **Point 10:** (1.4988, 4.7500)

4. Visual Field Mean Deviation (VF MD) Differences

Shapiro-Wilk Test Validation: W = 0.9457, P = 0.6432 (Normally Distributed - N = 9 Complete Paired Data Pairs)

- **Point 1:** (-1.4457, -3.4200)
- **Point 2:** (-0.9167, -2.4700)
- **Point 3:** (-0.5638, -1.3000)
- **Point 4:** (-0.2709, -0.1900)
- **Point 5:** (0.0000, 0.0100)
- **Point 6:** (0.2709, 0.2600)
- **Point 7:** (0.5638, 2.3100)
- **Point 8:** (0.9167, 3.2200)
- **Point 9:** (1.4457, 6.8300)

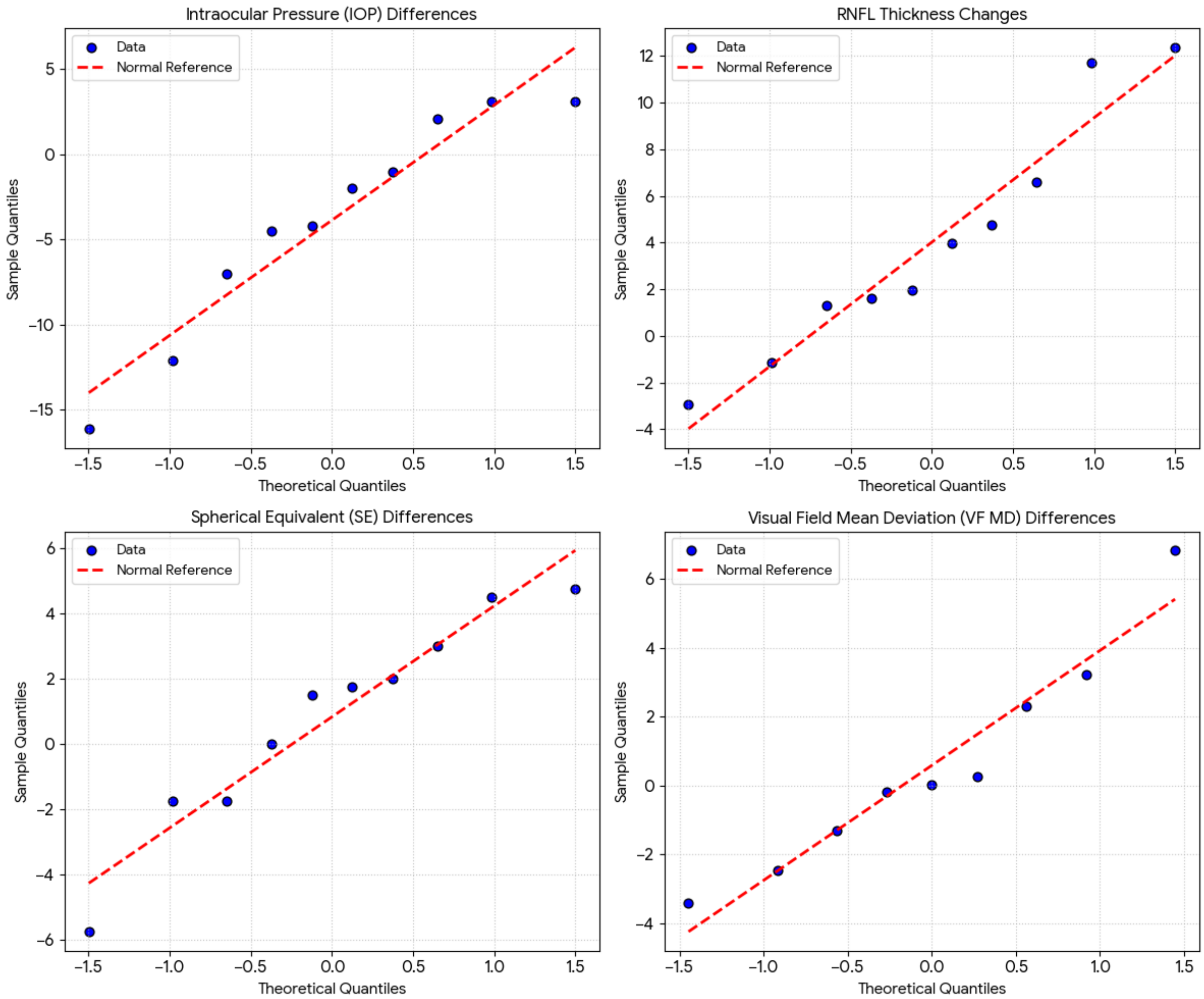

Supplement: Supplementary file 1 [file DataSheet1.pdf]
