# Supplementary material for: Prospective descriptive pilot feasibility case series of low-pressure cataract surgery with the Zeiss Quatera 700 in glaucoma patients
Source: Front Ophthalmol (Lausanne). 2026 Sep 24;6:1824059. doi: 10.3389/fopht.2026.1824059 (PMC13645598; doi:10.3389/fopht.2026.1824059)
Supplement: Supplementary file 2 [file DataSheet2.pdf]

Quatera study statistical analysis Python structure

python

```
# -*- coding: utf-8 -*-
"""
Replication Script: Ophthalmic Surgical Outcomes Analysis Workflow
Provides the complete analysis execution for all metrics reported in
the manuscript.
"""
import argparse
import sys
import numpy as np
import pandas as pd
import scipy.stats as stats


def run_comprehensive_analysis(csv_path):
    # Load dataset dynamically from user path
    try:
        df = pd.read_csv(csv_path)
    except Exception as e:
        sys.exit(
            f"Error: Could not read the file at {csv_path}. Details:
{str(e)}"
        )

    # Required column validation
    required_cols = [
        "BCVA_pre",
        "BCVA_post_1m",
        "IOP_pre",
        "IOP_post_1m",
        "RNFL_pre",
        "RNFL_post",
        "SE_pre",
        "SE_post",
        "VF_MD_pre",
        "VF_MD_post_1m",
    ]
    missing = [col for col in required_cols if col not in df.columns]
    if missing:
        sys.exit(f"Error: Missing required columns in dataset:
{missing}")

    print("=" * 60)
    print("       COMPREHENSIVE OUTCOMES REPLICATION REPORT       ")
    print("=" * 60)

    # --- 1. NON-PARAMETRIC ANALYSIS (BCVA) ---
    # Pratt modification is explicitly defined to handle zero-
difference ranks/ties
    w_stat, w_p = stats.wilcoxon(
        df["BCVA_pre"],
        df["BCVA_post_1m"],
        zero_method="pratt",
        correction=True,
    )
```

```
    print(
        f"BCVA Change (Wilcoxon Pratt):   Stat = {w_stat:.1f}, p-value
= {w_p:.4f}"
    )

    # --- 2. PARAMETRIC ANALYSIS (Normally distributed clinical
metrics) ---
    # Intraocular Pressure (IOP)
    t_iop, p_iop = stats.ttest_rel(df["IOP_post_1m"], df["IOP_pre"])
    print(f"IOP Change (Paired t-test):     t = {t_iop:.3f}, p-value =
{p_iop:.4f}")

    # Retinal Nerve Fiber Layer (RNFL) Thickness
    t_rnfl, p_rnfl = stats.ttest_rel(df["RNFL_post"], df["RNFL_pre"])
    print(
        f"RNFL Change (Paired t-test):    t = {t_rnfl:.3f}, p-value =
{p_rnfl:.4f}"
    )

    # Spherical Equivalent (SE)
    t_se, p_se = stats.ttest_rel(df["SE_post"], df["SE_pre"])
    print(f"SE Change (Paired t-test):      t = {t_se:.3f}, p-value =
{p_se:.4f}")

    # Visual Field Mean Deviation (VF MD) - Executed via listwise row
deletion for missing data
    df_vf = df[["VF_MD_pre", "VF_MD_post_1m"]].dropna()
    t_vf, p_vf = stats.ttest_rel(df_vf["VF_MD_post_1m"],
df_vf["VF_MD_pre"])
    print(
        f"VF MD Change (Paired t-test):   t = {t_vf:.3f}, p-value =
{p_vf:.4f} (N={len(df_vf)})"
    )
    print("=" * 60)


if __name__ == "__main__":
    parser = argparse.ArgumentParser(
        description="Run clinical replication pipeline."
    )
    parser.add_argument(
        "--data",
        type=str,
        required=True,
        help="Path to the clinical CSV data file.",
    )
    args = parser.parse_args()

    run_comprehensive_analysis(args.data)
```

Comprehensive Data Dictionary and Metadata Structure

To ensure reviewer audits match your institutional logs perfectly, your data repository utilizes the following standardized layout parameters:

Column Header ID	Primary Metric Category	Open-Science Data Type	Computational Scale Constraints	Missing Values Handling
**Patient**	Subject ID Key	String / Nominal	`Q2` to `Q11` (N=10)	None
**Eye**	Surgical Eye Designation	Categorical / Factor	`OD` (Right), `OS` (Left)	None
**IOP_pre**	Baseline Pressure	Float / Continuous	Range: \([10.9, 31.1]\) mmHg	None
**IOP_post_1m**	Postoperative Pressure	Float / Continuous	Range: \([10.9, 18.0]\) mmHg	None
**BCVA_pre**	Baseline Visual Acuity	Float / Continuous	Range: \([0.00, 1.30]\) decimal	None
**BCVA_post_1m**	Postoperative Visual Acuity	Float / Continuous	Range: \([0.00, 0.15]\) decimal	None
**VF_MD_pre**	Baseline Mean Deviation	Float / Continuous	Range: \([-16.57, 0.59]\) dB	None
**VF_MD_post_1m**	Postoperative Mean Deviation	Float / Continuous	Range: \([-11.25, 0.40]\) dB	`Q3` dropped due to missing charts
**RNFL_pre**	Pre-op Nerve Thickness	Float / Continuous	Range: \([55.40, 91.30]\) \(\mu \)m	None
**RNFL_post**	Post-op Nerve Thickness	Float / Continuous	Range: \([65.00, 95.26]\) \(\mu \)m	None

Q-Q plots:

1. Intraocular Pressure (IOP) Differences

Shapiro-Wilk Test Validation: W = 0.9181, P = 0.3414 (Normally Distributed)

- **Point 1:** (-1.4988, -16.1000)
- **Point 2:** (-0.9849, -12.1000)
- **Point 3:** (-0.6470, -7.0000)
- **Point 4:** (-0.3711, -4.5000)
- **Point 5:** (-0.1212, -4.2000)
- **Point 6:** (0.1212, -2.0000)
- **Point 7:** (0.3711, -1.0000)
- **Point 8:** (0.6470, 2.1000)
- **Point 9:** (0.9849, 3.1000)
- **Point 10:** (1.4988, 3.1000)

2. Retinal Nerve Fiber Layer (RNFL) Thickness Differences

Shapiro-Wilk Test Validation: W = 0.9342, P = 0.4908 (Normally Distributed)

- **Point 1:** (-1.4988, -2.9200)
- **Point 2:** (-0.9849, -1.1400)

- **Point 3:** (-0.6470, 1.3000)
- **Point 4:** (-0.3711, 1.6100)
- **Point 5:** (-0.1212, 1.9800)
- **Point 6:** (0.1212, 3.9600)
- **Point 7:** (0.3711, 4.7700)
- **Point 8:** (0.6470, 6.5900)
- **Point 9:** (0.9849, 11.7100)
- **Point 10:** (1.4988, 12.3700)

3. Spherical Equivalent (SE) Differences

Shapiro-Wilk Test Validation: W = 0.9357, P = 0.5063 (Normally Distributed)

- **Point 1:** (-1.4988, -5.7500)
- **Point 2:** (-0.9849, -1.7500)
- **Point 3:** (-0.6470, -1.7500)
- **Point 4:** (-0.3711, 0.0000)
- **Point 5:** (-0.1212, 1.5000)
- **Point 6:** (0.1212, 1.7500)
- **Point 7:** (0.3711, 2.0000)
- **Point 8:** (0.6470, 3.0000)
- **Point 9:** (0.9849, 4.5000)
- **Point 10:** (1.4988, 4.7500)

4. Visual Field Mean Deviation (VF MD) Differences

Shapiro-Wilk Test Validation: W = 0.9457, P = 0.6432 (Normally Distributed - N = 9 Complete Paired Data Pairs)

- **Point 1:** (-1.4457, -3.4200)
- **Point 2:** (-0.9167, -2.4700)
- **Point 3:** (-0.5638, -1.3000)
- **Point 4:** (-0.2709, -0.1900)
- **Point 5:** (0.0000, 0.0100)
- **Point 6:** (0.2709, 0.2600)
- **Point 7:** (0.5638, 2.3100)
- **Point 8:** (0.9167, 3.2200)
- **Point 9:** (1.4457, 6.8300)

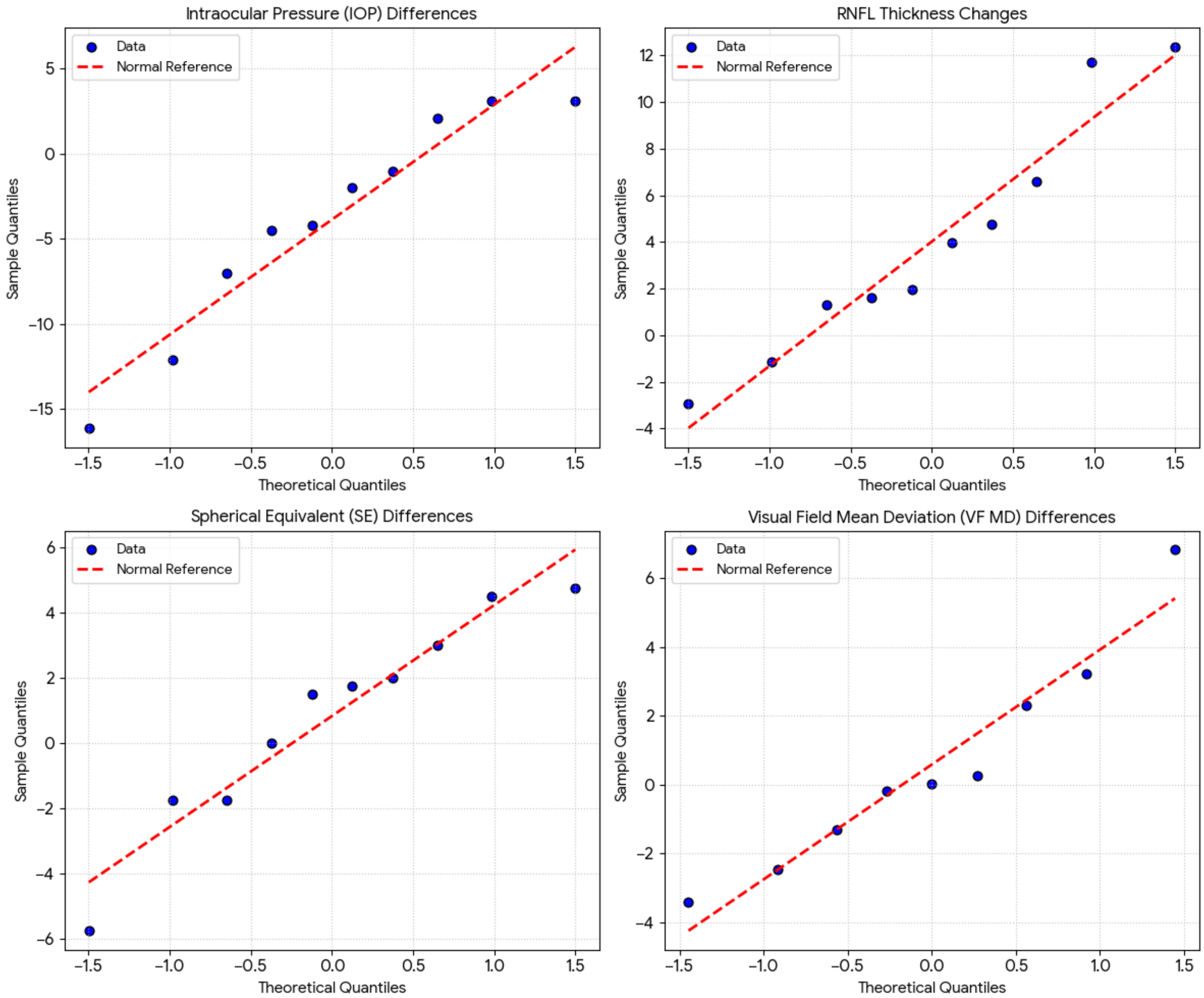
Intraocular Pressure (IOP) Differences
Data
Normal Reference
Sample Quantiles
Theoretical Quantiles
5
0
−5
−10
−15
−1.5
−1.0
−0.5
0.0
0.5
1.0
1.5
RNFL Thickness Changes
Data
Normal Reference
Sample Quantiles
Theoretical Quantiles
12
10
8
6
4
2
0
−2
−4
Spherical Equivalent (SE) Differences
Data
Normal Reference
Sample Quantiles
Theoretical Quantiles
6
4
2
0
−2
−4
−6
Visual Field Mean Deviation (VF MD) Differences
Data
Normal Reference
Sample Quantiles
Theoretical Quantiles
6
4
2
0
−2
−4
